# The Cardiac Care Bridge transitional care program for the management of older high-risk cardiac patients: An economic evaluation alongside a randomized controlled trial

**DOI:** 10.1371/journal.pone.0263130

**Published:** 2022-01-27

**Authors:** Lotte Verweij, Adrianne C. M. Petri, Janet L. MacNeil-Vroomen, Patricia Jepma, Corine H. M. Latour, Ron J. G. Peters, Wilma J. M. Scholte op Reimer, Bianca M. Buurman, Judith E. Bosmans

**Affiliations:** 1 Centre of Expertise Urban Vitality, Faculty of Health, Amsterdam University of Applied Sciences, Amsterdam, The Netherlands; 2 Department of Cardiology, Amsterdam UMC, University of Amsterdam, Amsterdam, The Netherlands; 3 Department of Internal Medicine, Section of Geriatric Medicine, Amsterdam UMC, University of Amsterdam, Amsterdam, The Netherlands; 4 Research Group Chronic Diseases, HU University of Applied Sciences Utrecht, Utrecht, The Netherlands; 5 Department of Health Sciences, Faculty of Science, Amsterdam Public Health Research Institute, Vrije Universiteit Amsterdam, Amsterdam, The Netherlands; Kurume University School of Medicine, JAPAN

## Abstract

**Objective:**

To evaluate the cost-effectiveness of the Cardiac Care Bridge (CCB) nurse-led transitional care program in older (≥70 years) cardiac patients compared to usual care.

**Methods:**

The intervention group (n = 153) received the CCB program consisting of case management, disease management and home-based cardiac rehabilitation in the transition from hospital to home on top of usual care and was compared with the usual care group (n = 153). Outcomes included a composite measure of first all-cause unplanned hospital readmission or mortality, Quality Adjusted Life Years (QALYs) and societal costs within six months follow-up. Missing data were imputed using multiple imputation. Statistical uncertainty surrounding Incremental Cost-Effectiveness Ratios (ICERs) was estimated by using bootstrapped seemingly unrelated regression.

**Results:**

No significant between group differences in the composite outcome of readmission or mortality nor in societal costs were observed. QALYs were statistically significantly lower in the intervention group, mean difference -0.03 (95% CI: -0.07; -0.02). Cost-effectiveness acceptability curves showed that the maximum probability of the intervention being cost-effective was 0.31 at a Willingness To Pay (WTP) of €0,00 and 0.14 at a WTP of €50,000 per composite outcome prevented and 0.32 and 0.21, respectively per QALY gained.

**Conclusion:**

The CCB program was on average more expensive and less effective compared to usual care, indicating that the CCB program is dominated by usual care. Therefore, the CCB program cannot be considered cost-effective compared to usual care.

## Introduction

Cardiac disease is the leading cause of hospitalization and mortality in older individuals and leads to substantial healthcare costs [1, 2]. Approximately 14% of total US healthcare costs [1] and approximately 12% of the total healthcare expenditure in the Netherlands are caused by cardiac disease and the majority of costs is incurred in older individuals [3]. After hospitalization for cardiac disease, up to 25% of older cardiac patients are readmitted within the first six months [4, 5]. Geriatric conditions lead to physical and cognitive limitations, thereby complicating medical treatment and care during and after discharge. This increases the risk of adverse outcomes such as hospital readmission [6] and contribute to high healthcare costs [7]. There is increasing evidence that a large proportion of costly readmissions can be prevented [8].

Transitional care interventions have the potential to reduce the risk of readmission and mortality [9–11]. However, in cardiac patients the evidence is not unequivocal [9, 12–14]. The Cardiac Care Bridge transitional care program (CCB program) was developed to reduce hospital readmission and mortality in older (≥70 years) cardiac patients at high risk of readmission and mortality [15, 16]. This nurse-coordinated intervention combined case management, disease management and home-based rehabilitation in the transition of care. The aim of the current study is to assess the cost-effectiveness of the CCB program compared to usual care from a societal perspective, within six months after randomization among older (≥70 years) cardiac patients at high risk of readmission and mortality.

## Materials and methods

### Design

A cost-effectiveness analysis of the CCB program was performed alongside the CCB randomized controlled trial from a societal perspective. The study protocol was approved by the Medical Ethics Committee of the Amsterdam University Medical Centre (Protocol ID: MEC2016_024) and registered in the trial registration: NTR6316 (http://www.trialregister.nl). All participants provided written informed consent. This manuscript was designed according to the CHEERS criteria, see S1 Table [17].

### Participants

The CCB multi-centre randomized trial was conducted between June 2017 and March 2019 in six hospitals in and surrounding Amsterdam, the Netherlands [15]. In total, 306 older (≥70 years) hospitalized cardiac patients at high risk of readmission and mortality were included. Patients were eligible for inclusion if they were at high risk according to the Dutch Safety Management System (DSMS) screening on malnutrition, fall risk, delirium and functional impairment, or if patients had an unplanned hospital admission within six months prior to the index admission and were discharged home. The DSMS-score ranges between 0–4 and patients were considered at high risk with a DSMS-score ≥2 in patients aged 70–79 years or DSMS-score ≥1 in patients aged ≥80 years [15].

### Randomization

Within 72 hours of hospitalization, eligible patients were asked to participate in the randomized trial by cardiac research nurses [15, 16]. After providing informed consent, a comprehensive geriatric assessment (CGA) was conducted with all participants. Subsequently, participants were randomized to the intervention or usual care group by a web-based program to ensure allocation concealment (Research Manager, https://my-researchmanager.com/en/). Participants were blinded to their group allocation according to a postponed informed consent procedure [18].

### Intervention

In brief, the CCB program included three phases (clinical, discharge and post-clinical phase) and consisted of three core components (case management, disease management and home-based cardiac rehabilitation) [15, 16]. In the clinical phase, the cardiac research nurses developed an integrated care plan together with participants, based on cardiac and geriatric conditions as assessed by the CGA, and consulted other disciplines based on indication. In the discharge phase, community nurses visited participants in hospital prior to discharge to receive a face-to-face handover from the cardiac research nurse and to meet participants. The community-based physical therapist received a written handover and the discharge date to organize home-based cardiac rehabilitation. After discharge, the participants received four home visits from the community nurse which were focussed on medication reconciliation, evaluation of the health status and the integrated care plan, and topics related to lifestyle. The community nurse was in close contact with an affiliated pharmacist for medication reconciliation and with the community-based physical therapist who performed up to nine home-based cardiac rehabilitation sessions.

### Usual care

Standard primary care was provided in both the intervention and the usual care group. During hospitalization, participants received care as usual from their treating cardiologist. After discharge, participants received outpatient care from a cardiologist and cardiac nurse specialist according to the national cardiovascular guidelines [16, 19]. The treating cardiologist referred participants to outpatient or centre-based cardiac rehabilitation programs on indication. For non-cardiovascular problems, the general practitioner is the primary healthcare provider. In the Netherlands, basic healthcare insurance is obliged in all citizens. It includes coverage of primary care visits, hospital outpatient visits, hospitalizations, and prescribed medication. Supplementary insurance can be purchased and includes e.g., physical therapy and other paramedical services.

### Outcomes

The primary outcome of the CCB study was the composite of first all-cause unplanned hospital readmission or mortality within six months follow-up. These outcomes were assessed by medical files of participating hospitals, the Dutch National Personal Records Database and self-reported information during follow-up [15, 16].

Health-related Quality of Life (HQoL) was evaluated at six months follow-up by using the 5-level EuroQol-5D questionnaire (EQ5D-5L) [20]. Subsequently, the Dutch EQ-5D-5L tariff (based on the Dutch general society) was used to convert the EQ-5D-5L health states into utilities [21]. Finally, QALYs were calculated by multiplying the time subjects spent by the utilities of that health state. The changes in utilities between two measurement points were assumed linear.

Healthcare utilization and costs were measured from a societal perspective which means that all costs, including informal and healthcare costs, were included in the analyses (see Table 1) [22]. Healthcare utilization at three and six months follow-up, was collected by use of an extended version of The Older Persons and Informal Caregivers Survey—Minimum Data Set (TOPIC-MDS) and included the length of hospital admissions, the number of emergency visits, the number of days in residential care, the number of days receiving day care, the number of general practitioner consultations, pharmacist consultations, hours of received personal care and home nursing, hours of received physical therapy and duration of outpatient rehabilitation or hospital-based rehabilitation [23]. These data were self-reported and supplemented with information from the hospital medical files. Informal care hours were self-reported by the informal caregiver. To convert healthcare utilization into healthcare costs, Dutch standard costs were multiplied by the volumes of utilization of these units [24]. All prices were converted into prices for the year 2018 using consumer price indices, see Table 1 [25].

**Table 1:** Healthcare costs (€) used in the cost-effectiveness analysis.

Healthcare utilization		Volume	Costs^a^
**Primary care**			
General practitioner consultation		Visit	34.34
Community pharmacist medication reconciliation		Visit	49.33
Home care			
	Community nursing	Hour	75.97
	Personal care	Hour	52.04
	Domestic care at home	Hour	23.53
Care hotel (in nursing home)		Day	174.83
Day-care		Day	139.45
Physical therapy		Visit	34.34
Physical therapy, home visit		Visit	45.77
**Secondary care**			
Emergency room		Visit	269.52
Hospital admission		Day	495.34
Hospital ICU admission		Day	2096.89
Outpatient clinic		Visit	94.70
Rehabilitation			
	Institutional	Day	478.69
	Outpatient cardiac rehabilitation	Hour	156.54
Residential and nursing home care		Day	174.83
**Informal care**			
Voluntary care, housekeeping, practical caregiver support		Hour	14.32

^a^Prices are obtained from the Dutch manual for cost-analysis in healthcare research [24]. Subsequently, prices per categories were indexed to the reference year 2018 by using a consumer price index [25]. The price of the pharmacist consultation is based on the Dutch guideline ‘Generieke kosten medicatiebeoordeling’ (General costs medication reconciliation) [26].

To calculate the intervention costs, the intervention components were valued with Dutch standard costs according to the Dutch guidelines using a bottom-up micro-costing approach [25]. In addition, the time needed to perform a baseline assessment, to develop an integrated care plan and to arrange the home-based intervention, was based on an average time-investment estimation within the CCB study protocol and was valued using standardized salary costs, see Table 2 [16].

**Table 2:** CCB intervention costs (€).

	Minutes per participant	Costs per hour^a^	Total CCB costs
**Secondary care**			
Comprehensive geriatric assessment	100	19.29	32.15
Integrated care plan	30	19.29	9.64
Consultation geriatrician	15	117.59	29.39
Face-to-face handover cardiac nurse	30	19.29	9.64
**Primary care**			
Community nurse (home) visits, including in hospital face-to-face handover^b^	5–6 visits	NA	241.00
Pharmacist medication reconciliation^c^	20	147.48	49.33
Home-based cardiac rehabilitation (9 sessions)	285	45.77	411.93

^a^Prices are obtained from the Dutch manual for cost-analysis in healthcare research [24]. Subsequently, prices per categories were indexed to the reference year 2018 using a consumer price index [25].

^b^Community nurse visits: 1–9 visits ≤ 3 months category frail / chronically ill, standard price.

^c^The price of the pharmacist consultation is based on the Dutch guideline ‘Generieke kosten medicatiebeoordeling’ (General costs medication reconciliation) [26].

### Missing data

Missing observations in cost and effect data were imputed using multiple imputation by chained equations (MICE) with predictive mean matching [27, 28]. The imputation model included variables that were related to missingness or the outcome, and all variables included in the analysis models, see S2 Table. Based on the loss of efficiency (fraction of missing information/*m*≤0.05), ten imputed datasets were needed [28]. These imputed datasets were analysed separately, after which the results were pooled using Rubin’s rules [29].

### Statistical analysis

All analyses were performed according to the intention-to-treat principle. Baseline characteristics were presented as mean with standard deviation (SD), median with interquartile range (IQR) or number with percentage. Seemingly unrelated regression (SUR) was performed to estimate cost and effect differences adjusted for confounders [30]. Variables were considered to be a confounder if their inclusion resulted in a ≥10% change in the beta-coefficient, and included sex, cardiovascular diagnosis and geriatric conditions: malnutrition, falling, delirium, functional impairment and cognitive status Mini-Mental State Examination-score [16]. Cost data generally have a highly skewed distribution due to many patients with low costs and few patients with (very) high costs, and no possibility of negative values. Therefore, statistical uncertainty was estimated by bootstrapping the SUR models using 5000 replications.

Incremental cost‐effectiveness ratios (ICERs) were calculated by dividing the difference in total costs between the intervention group and the usual care group by the difference in the composite outcome (first readmission or mortality) for the cost-effectiveness analysis (CEA) and QALYs for the cost-utility analysis (CUA). Statistical uncertainty surrounding the ICERs was presented by showing the bootstrapped cost-effect pairs in cost-effectiveness planes. In a cost-effectiveness plane, the difference in effects between the intervention and usual care group is plotted on the x axis and the difference in costs on the y axis. Cost-effectiveness acceptability curves (CEAC) were estimated, showing the probability that the intervention is cost-effective compared to control for all possible values of the willingness to pay (WTP) threshold. The WTP threshold represents the amount of money that society is willing to pay to obtain one unit of effect extra [31].

Two sensitivity analyses were performed. First, the main analysis was repeated without adjustment for confounders. Second, analyses were performed from a healthcare perspective in which only healthcare costs were included.

IBM SPSS version 26.0 0 (SPSS Inc., Chicago, IL, USA) and Stata Statistical Software: Release 16 (College Station, TX: StataCorp LP) were used in the data analyses.

## Results

In total, 306 participants were included in the CCB study and were randomly allocated to the intervention (n = 153) or the usual care group (n = 153). Table 3 presents the baseline characteristics. The only baseline difference found, was a higher risk of delirium in the intervention group compared to the usual care group, 61.4% and 50.3% (p = 0.049) respectively.

**Table 3:** Baseline characteristics.

	Intervention n = 153	Usual care n = 153
**Socio-demographics **		
Male	70 (45.8)	86 (56.2)
Age, years	82.5 ± 6.1	82.3 ± 6.5
Cohabitating	66 (43.1)	68 (44.4)
**Disease related characteristics **		
Hospital admission ≤ 6 months of index hospitalization	66 (43.1)	73 (47.7)
Cardiac diagnosis on admission		
- Heart failure	86 (56.2)	91 (59.5)
- Acute Coronary Syndrome	19 (12.4)	24 (15.7)
- Other	48 (31.4)	38 (24.8)
Charlson Comorbidity index	3 [1–4]	3 [1–4]
**Geriatric conditions**		
(Risk of) delirium^a^	94 (61.4)	77 (50.3)
Fall risk (fall ≤ 6 months)	67 (43.8)	78 (51.0)
Functional impairment (Katz-6, score ≥2)	65 (42.5)	54 (35.3)
(Risk of) malnutrition (SNAQ)	57 (37.3)	43 (28.1)
Cognitively impaired, MMSE 15–23	47 (30.7)	48 (31.4)

N (%), mean ± standard deviation (SD), median with interquartile range [IQR].

^a^Assessment of 1. cognitive impairment; 2. help with self-care ≤ 24 hours; 3. a previously delirium (≥1 point = at risk).

Abbreviations: MMSE mini-mental state examination, SNAQ short nutritional assessment questionnaire.

**Fig 1:**
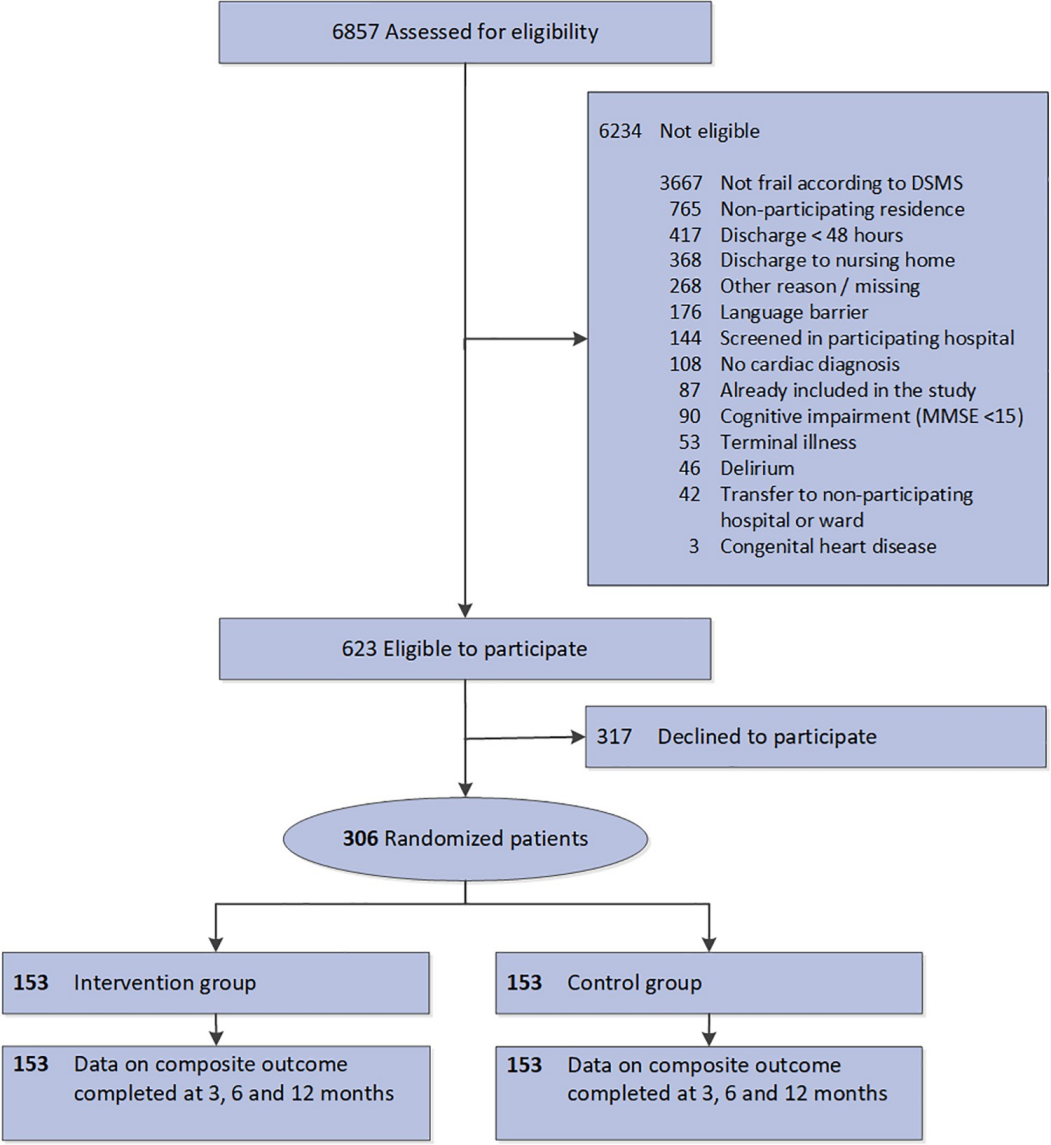
Flowchart.

Complete outcome data on the composite outcome were available from all participants, see Fig 1. Data on costs over six months follow-up were complete in 75 (49.0%) intervention participants and in none of the participants in the usual care group (see S3 Table). In total, 227 participants (74.2%) had complete data on QALYs at six months follow-up, of whom 119/153 participants (77.8%) in the intervention group and 108/153 participants (70.6%) in the usual care group. Between group differences were tested in participants with and without missing data on costs and no significant differences were found.

### Outcomes

#### Primary outcomes

Table 4 shows the unadjusted mean outcomes over six months follow-up. In the intervention group, the proportion of participants with the primary composite outcome of readmission or mortality was 54% compared to 48% in the usual care group (risk difference (RD), 6% (95% confidence interval (CI) -5%; 18%). The mean difference in QALYs between the intervention (mean 0.35, SD 0.14) and usual care group (mean 0.38, SD 0.14) was -0.03 (95% CI: -0.07; -0.02).

**Table 4:** Unadjusted mean costs (€) and effects over 6 months follow-up after multiple imputation.

	Intervention group (N = 153)	Usual care group (N = 153)	Mean difference	95% CI
**Outcomes**				
Readmission or mortality	0.54 (0.50)	0.48 (0.50)	0.06	-0.05; 0.18
QALY	0.35 (0.14)	0.38 (0.14)	-0.03	-0.07; -0.02
**Costs**				
Healthcare costs, primary care	8348 (18030)	8501 (21338)	-153	-1534; 1228
Healthcare costs, secondary care	5336 (8139)	5256 (7772)	-80	-468; 628
Informal care costs	2445 (9178)	962 (3407)	1483	1009; 1956
**Total costs from a societal perspective, including all costs**	16126 (23288)	14833 (23438)	1294	-343; 2931
**Total costs from a healthcare perspective, primary and secondary care costs**	13717 (19425)	13873 (22631)	-155	-1630; 1320

Mean, standard deviation (SD), confidence interval (CI).

#### Costs

Table 4 shows the crude mean costs over six months follow-up after multiple imputation. There was no difference in total societal costs between groups. Informal care costs were significantly higher in the intervention versus the usual care group. Primary care costs were the largest cost driver in both groups.

#### Cost-effectiveness

**Fig 2:**
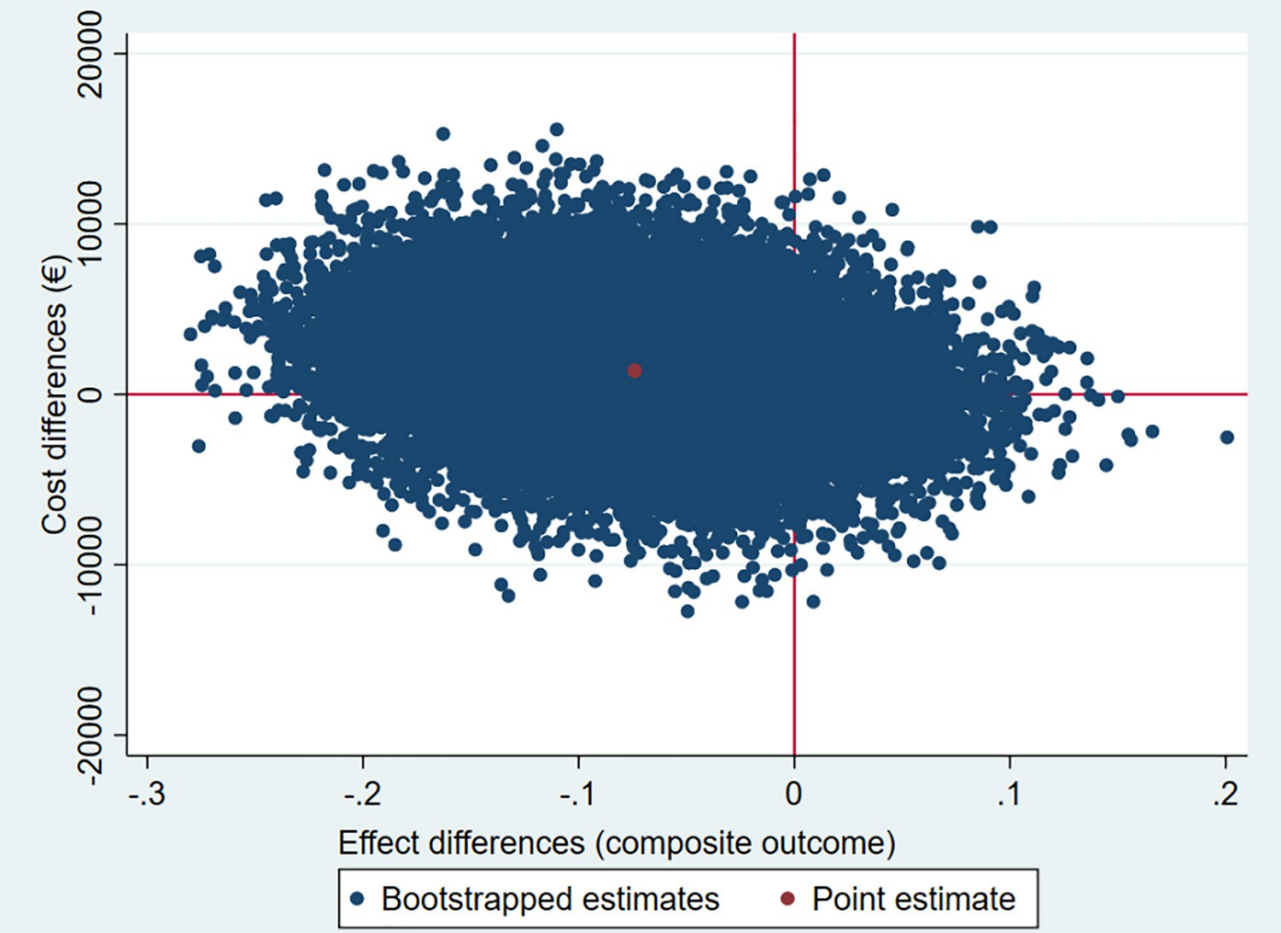
Cost-effectiveness plane for estimated readmission or mortality comparing the intervention group with the usual care group. North-East quadrant: more effective and more expensive, North-West quadrant: less effective and more expensive, South-West quadrant: less effective and less expensive, South-East quadrant: more effective and less expensive.

**Fig 3:**
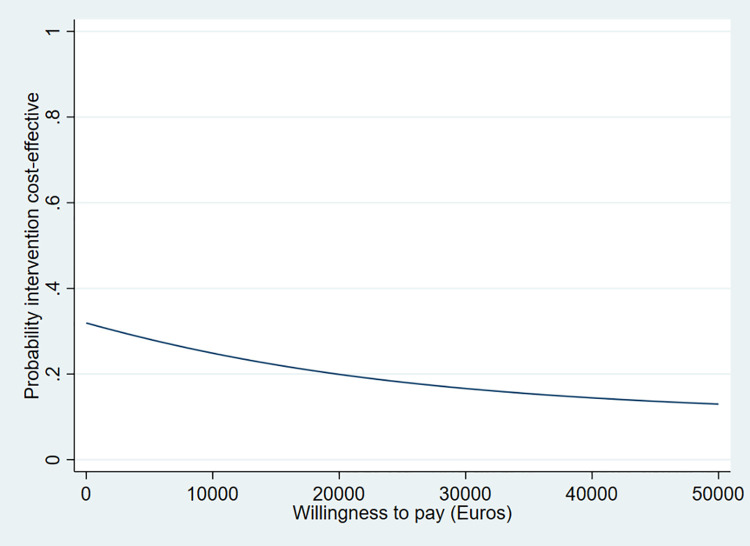
Cost-effectiveness acceptability curve for readmission or mortality. The acceptability curve shows the probability that the intervention is cost-effective (y-axis) compared to usual care over a range of ‘willingness to pay’ (WTP) values (x-axis). The WTP indicates the value that one is willing to pay for one unit of effect.

The results of the CEA are presented in Table 5, and Figs 2 and 3. Table 5 and Fig 2 show that the ICER and 64% of the cost-effect pairs are in the northwest quadrant of the CE-plane, indicating that the intervention is on average more expensive and less effective (higher incidence of the composite outcome of first readmissions and mortality) compared to usual care. The CEA curve in Fig 3 shows that the probability of the intervention being cost-effective compared to the usual care group was 31% when the WTP is €0 per prevented case of readmission or mortality. This probability decreases to 14% when the WTP is €50,000 per prevented case of readmission or mortality.

**Table 5:** Differences in readmission or mortality, QALYs and costs in €, ICERs, distribution of bootstrapped cost-effect pairs over the quadrants of the CE-plane, and the probability of cost-effectiveness at different ceiling ratios.

	Cost Δ (95% CI)	Effect Δ (95% CI)	ICER	CE-plane	CE-plane	CE-plane	CE-plane	Probability that CCB-intervention is CE at WTP		
* *				NE	SE	SW	NW	WTP = €0	WTP = €30,000	WTP = €50,000
**Main outcome: societal perspective adjusted for confounding**										
Composite outcome of readmission or mortality at 6 months	1404 (-4050;6648)	-0.074 (-0.184;0.036)	-22,903	5%	5%	26%	64%	31%	18%	14%
QALYs	1346 (-4104;6554)	-0.025 (-0.059;0.008)	-55,190	4%	3%	28%	65%	32%	24%	21%
***Sensitivity analysis*: *societal perspective***										
Composite outcome	1435 (-3860;6551)	-0.065 (-0.177;0.046)	-24,458	7%	6%	24%	63%	31%	19%	17%
QALYs	1435 (-3826;6512)	-0.025 (-0.059;0.009)	-56,344	4%	4%	26%	66%	31%	24%	20%
***Sensitivity analyses*: *healthcare perspective***										
Composite outcome	-156 (-5339;4191)	-0.074 (-0.184;0.036)	-195	3%	7%	45%	45%	52%	28%	21%
QALYs	-208 (-5397;4121)	-0.025 (-0.059;0.008)	1613	2%	5%	48%	45%	54%	42%	38%

Abbreviations and explanation: NE quadrant: more effective and more expensive, SE quadrant: more effective and less expensive, SW quadrant: less effective and less expensive, NW quadrant: less effective and more expensive. CCB: Cardiac Care Bridge, CE: cost-effective, WTP: willingness to pay, QALY: quality adjusted life years.

#### Cost-utility

**Fig 4:**
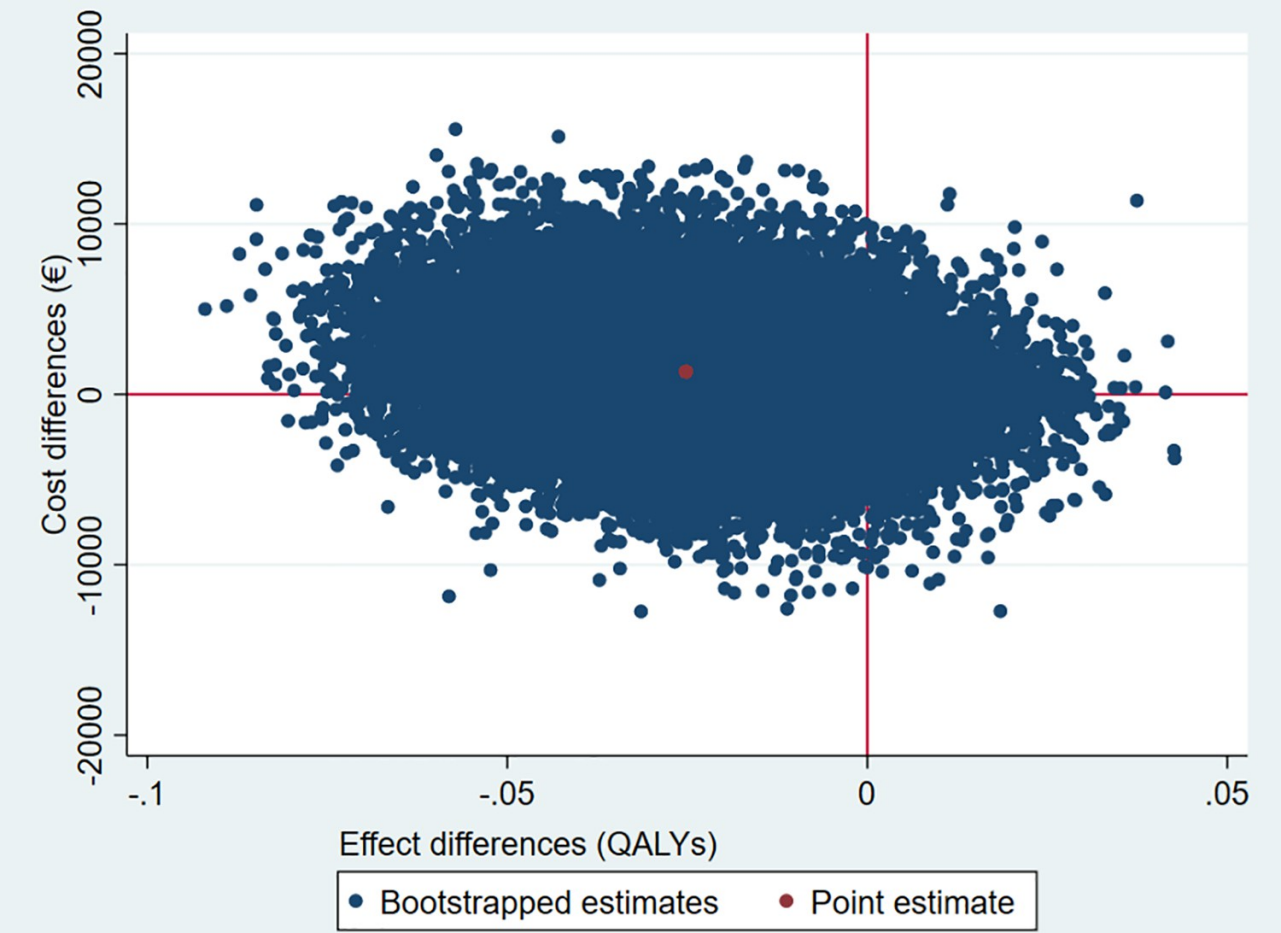
Cost-effectiveness plane for QALYs comparing the intervention group to the usual care group. North-East quadrant: more effective and more expensive, North-West quadrant: less effective and more expensive, South-West quadrant: less effective and less expensive, South-East quadrant: more effective and less expensive.

**Fig 5:**
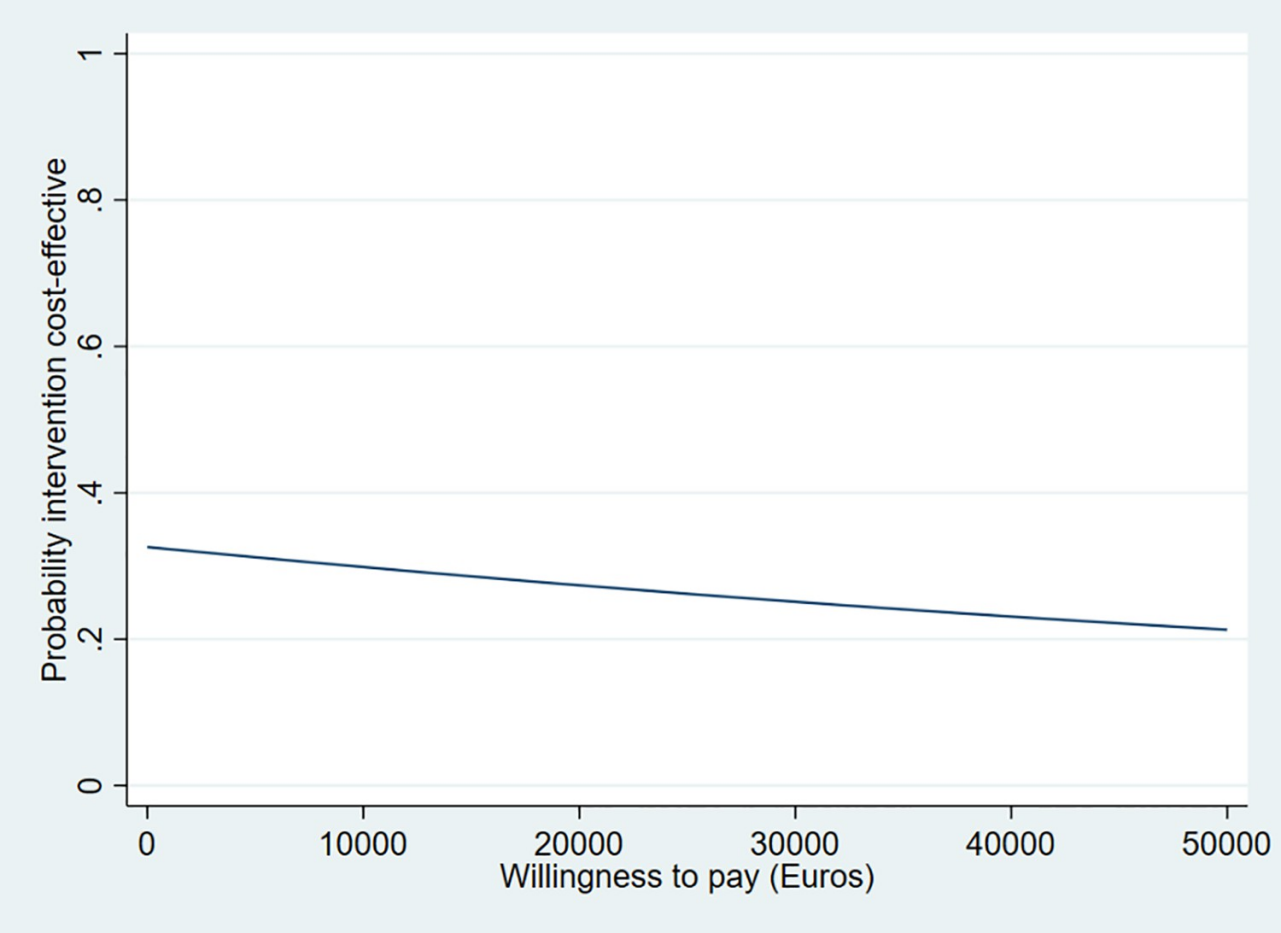
Cost-effectiveness acceptability curve for QALYs. The acceptability curve shows the probability that the intervention is cost-effective on QALYs (y-axis) compared to usual care over a range of ‘willingness to pay’ values (x-axis). The WTP indicates the value that one is willing to pay for one unit of effect.

The results of the CUA are shown in Table 5, Figs 4 and 5. Table 5 and Fig 4 show that the ICER and 65% of the cost-effect pairs are in the northwest quadrant of the CE-plane, indicating that the intervention was more expensive and less effective (less QALYs) compared to usual care. In Fig 5, the CEA curve shows that the probability that the intervention is cost-effective compared to the usual care group (on QALYs) was 32% when the WTP is €0 per QALY gained. This probability decreases to 21% when the WTP is €50,000 per QALY.

#### Sensitivity analyses

In Table 5, the results of the sensitivity analyses for the CEA and CUA are also presented. Results of the sensitivity analyses of the societal perspective as well as analyses from healthcare perspective, were in line with the results from the main analysis.

## Discussion

In this study, no significant differences were found on the composite outcome of first unplanned readmission or mortality and total societal costs. In addition, the number of QALYs was significantly lower in the intervention group. The CCB program was on average more expensive and less effective than usual care, meaning that the CCB program was dominated by usual care.

Although our study is the first cost-effectiveness study of an intervention combining case management, disease management and home-based cardiac rehabilitation in the transition of care [15, 16], there are some previous studies on cost-effectiveness of nurse-led transitional care interventions in heart failure patients. For example, the systematic review of Bryant et al. [32] showed that such interventions had a favourable effect on outcomes such as rehospitalization and reduced costs in patients with heart failure compared to usual care. Other studies on nurse-led transitional care services, showed similar favourable outcomes and reduced costs, but did not report QALYs [14, 33]. The most likely explanation for the contrasting results regarding both costs and effects found in our study is that our study population was older (mean age 82 years) and more frail than in the previously published studies. Despite the lack of clinical effects, we considered it important to conduct a full economic evaluation, because there may still be a relevant impact on costs. Also, even when both cost and effect differences are not statistically significant, based on the joint uncertainty surrounding costs and effects there may be values of the ceiling ratio at which the intervention is considered cost-effective compared to usual care.

The CCB intervention was evaluated in a randomized controlled trial design and implemented on top of the usual care systems [16]. Although healthcare costs did not significantly differ between the intervention and usual care group, there was a statistically significant difference in informal care costs. It was part of the CCB protocol to involve informal caregivers in the process which may have resulted in higher overall informal caregiver support.

### Strengths and limitations

Several strengths are relevant to our study. First, data on readmissions and mortality were collected using both self-reported data and hospital and municipality records. This reduced the chance of recall bias and improved the validity of the data. Second, in order to estimate the costs of the CCB intervention, we used a bottom-up micro-costing approach which is a more precise method to estimate costs than a top-down costing approach [24]. Third, costs were measured from a societal perspective. This is the broadest approach possible and takes all costs into account regardless who pays for them [24]. This enables the identification of potential shifts in costs between budgets. For example, early discharge may reduce healthcare costs but may increase informal care costs. Finally, we performed a sensitivity analysis from a healthcare perspective. This perspective is used for decision making in many countries, such as for example the United Kingdom. Thus, it also allows for comparison of the results with cost-effectiveness studies from these countries [24].

Some aspects of our study warrant consideration. There was a high percentage of missing data on both costs and on HQoL. This missingness was probably caused by several factors, such as withdrawal from follow-up visits, recall problems and non-response from informal caregivers. Considering that people tend to underestimate their healthcare use [34] and the high age of the included participants, recall bias on healthcare use (i.e. other than hospital readmission) was probably present and may have led to an underestimation of costs in all participants. To reduce the chance of recall bias as much as possible, measurements were performed at both three and six months follow-up [35]. In this study, multiple imputation was used to impute missing data, since this is considered the most valid method to deal with missing data [36]. Baseline variables that were used as predictor variables for multiple imputation were carefully selected, based on their association with missingness or the outcome. Last, from the CCB process evaluation, it is known that the mean intervention fidelity rate was only 67%, which could have influenced the effect on the composite outcome and intervention costs [37]. However, we calculated the intervention costs from a standardized intervention cost price instead of a fidelity-based cost price based per individual which could have resulted in a slight overestimation of the actual intervention costs.

### Implications

Based on the current study results, the CCB program cannot be considered cost-effective compared to usual care. Considering the resources needed to implement such an intervention, we recommend against implementation of the intervention in clinical practice in its current form. Further research is needed to find suitable interventions to meet frail cardiac patients’ needs and to reduce adverse outcomes and costs, and increase HQoL.

## Conclusion

The CCB program was on average more expensive from a societal perspective and less effective compared to usual care, indicating that the CCB program is dominated by usual care. Therefore, the CCB program cannot be considered cost-effective compared to usual care.

## Supporting information

S1 Checklist CONSORT 2010 checklist of information to include when reporting a randomised trial*. (DOC)

S1 Table CHEERS checklist. (DOCX)

S2 Table Variables included in the imputation model. (DOCX)

S3 Table Missing values. (DOCX)

## References

[1] Benjamin EJ, Muntner P, Alonso A, Bittencourt MS, Callaway CW, Carson AP, et al. Heart Disease and Stroke Statistics-2019 Update: A Report From the American Heart Association. Circulation. 2019;139(10):e56–e528. doi: 10.1161/CIR.0000000000000659 30700139

[2] WHO. Top 10 causes of death https://www.who.int/data/gho/data/themes/topics/causes-of-death World Health Organisation; [Available from: http://www.who.int/en/news-room/fact-sheets/detail/the-top-10-causes-of-death.

[3] RIVM. Hart- en vaatziekten 2017 volksgezondheidenzorg.info/onderwerp/hart-envaatziekten/kosten/zorguitgaven Volksgezondheidenzorg; [Available from: https://www.volksgezondheidenzorg.info/onderwerp/hartfalen/kosten/zorguitgaven#node-zorguitgaven-hartfalen-naar-leeftijd-en-geslacht.

[4] Jepma P, Ter Riet G, van Rijn M, Latour CHM, Peters RJG, Scholte Op Reimer WJM, et al. Readmission and mortality in patients ≥70 years with acute myocardial infarction or heart failure in the Netherlands: a retrospective cohort study of incidences and changes in risk factors over time. Netherlands heart journal: monthly journal of the Netherlands Society of Cardiology and the Netherlands Heart Foundation. 2019;27(3):134–41. doi: 10.1007/s12471-019-1227-4 30715672 PMC6393584

[5] Krumholz HM, Lin Z, Keenan PS, Chen J, Ross JS, Drye EE, et al. Relationship between hospital readmission and mortality rates for patients hospitalized with acute myocardial infarction, heart failure, or pneumonia. Jama. 2013;309(6):587–93. doi: 10.1001/jama.2013.333 23403683 PMC3621028

[6] Bell SP, Patel N, Patel N, Sonani R, Badheka A, Forman DE. Care of older adults. Journal of geriatric cardiology: JGC. 2016;13(1):1–7. doi: 10.11909/j.issn.1671-5411.2016.01.019 26918006 PMC4753005

[7] Ribbink ME, van Seben R, Reichardt LA, Aarden JJ, van der Schaaf M, van der Esch M, et al. Determinants of Post-acute Care Costs in Acutely Hospitalized Older Adults: The Hospital-ADL Study. Journal of the American Medical Directors Association. 2019;20(10):1300–6.e1. doi: 10.1016/j.jamda.2019.03.013 31056452

[8] Stewart S. Financial aspects of heart failure programs of care. European journal of heart failure. 2005;7(3):423–8. doi: 10.1016/j.ejheart.2005.01.001 15718184

[9] Feltner C, Jones CD, Cene CW, Zheng ZJ, Sueta CA, Coker-Schwimmer EJ, et al. Transitional care interventions to prevent readmissions for persons with heart failure: a systematic review and meta-analysis. Ann Intern Med. 2014;160(11):774–84. doi: 10.7326/M14-0083 24862840

[10] Naylor MD, Shaid EC, Carpenter D, Gass B, Levine C, Li J, et al. Components of Comprehensive and Effective Transitional Care. Journal of the American Geriatrics Society. 2017;65(6):1119–25. doi: 10.1111/jgs.14782 28369722 PMC5497308

[11] Verhaegh KJ, MacNeil-Vroomen JL, Eslami S, Geerlings SE, de Rooij SE, Buurman BM. Transitional care interventions prevent hospital readmissions for adults with chronic illnesses. Health Aff (Millwood). 2014;33(9):1531–9. doi: 10.1377/hlthaff.2014.0160 25201657

[12] Meisinger C, Stollenwerk B, Kirchberger I, Seidl H, Wende R, Kuch B, et al. Effects of a nurse-based case management compared to usual care among aged patients with myocardial infarction: results from the randomized controlled KORINNA study. BMC geriatrics. 2013;13:115. doi: 10.1186/1471-2318-13-115 24168465 PMC3871021

[13] Van Spall HGC, Lee SF, Xie F, Oz UE, Perez R, Mitoff PR, et al. Effect of Patient-Centered Transitional Care Services on Clinical Outcomes in Patients Hospitalized for Heart Failure: The PACT-HF Randomized Clinical Trial. Jama. 2019;321(8):753–61. doi: 10.1001/jama.2019.0710 30806695 PMC6439867

[14] Van Spall HGC, Rahman T, Mytton O, Ramasundarahettige C, Ibrahim Q, Kabali C, et al. Comparative effectiveness of transitional care services in patients discharged from the hospital with heart failure: a systematic review and network meta-analysis. European journal of heart failure. 2017;19(11):1427–43. doi: 10.1002/ejhf.765 28233442

[15] Jepma P. VL, Buurman B.M., Terbraak M.S., Daliri S., Latour C.H.M., Riet ter G., et al. The nurse-coordinated cardiac care bridge transitional care programme: a randomised clinical trial. Age and Ageing. doi: 10.1093/ageing/afab146 2021:1–11. 34304264 PMC8581392

[16] Verweij L, Jepma P, Buurman BM, Latour CHM, Engelbert RHH, Ter Riet G, et al. The cardiac care bridge program: design of a randomized trial of nurse-coordinated transitional care in older hospitalized cardiac patients at high risk of readmission and mortality. BMC Health Serv Res. 2018;18(1):508. doi: 10.1186/s12913-018-3301-9 29954403 PMC6025727

[17] Husereau D, Drummond M, Petrou S, Carswell C, Moher D, Greenberg D, et al. Consolidated Health Economic Evaluation Reporting Standards (CHEERS)—explanation and elaboration: a report of the ISPOR Health Economic Evaluation Publication Guidelines Good Reporting Practices Task Force. Value in health: the journal of the International Society for Pharmacoeconomics and Outcomes Research. 2013;16(2):231–50. doi: 10.1016/j.jval.2013.02.002 23538175

[18] Boter H, van Delden JJ, de Haan RJ, Rinkel GJ. [A modified informed-consent procedure in which the complete information is given retrospectively: no objection from participating patients]. Ned Tijdschr Geneeskd. 2005;149(1):29–32. 15651501

[19] Cardiology DSf. Multidisciplinary guideline for cardiac Rehabilitation: Dutch Society for Cardiology 2011 [Available from: https://www.nvvc.nl/hr.

[20] Herdman M, Gudex C, Lloyd A, Janssen M, Kind P, Parkin D, et al. Development and preliminary testing of the new five-level version of EQ-5D (EQ-5D-5L). Qual Life Res. 2011;20(10):1727–36. doi: 10.1007/s11136-011-9903-x 21479777 PMC3220807

[21] Versteegh M, Vermeulen K, Evers S, de Wit G, Prenger R, Stolk E. Dutch Tariff for the Five-Level Version of EQ-5D. Value in health: the journal of the International Society for Pharmacoeconomics and Outcomes Research. 2016;19(4):343–52. doi: 10.1016/j.jval.2016.01.003 27325326

[22] Drummond M. Methods for the Economic Evaluation of Healthcare Programmes. Fourth ed: Oxford University Press; 2015.

[23] The Older Persons and Informal Caregivers Survey—Minimum Data Set (TOPIC-MDS) National Care for the Elderly Program [Available from: http://www.beteroud.nl/ouderen/topics-mds-database-vragenlijst.html.

[24] Hakkaart-van Roijen L. Kostenhandleiding. Methodologie van kostenonderzoek en referentieprijzen voor economische evaluaties in de gezondheidszorg.: Zorginstituut Nederland; 2015.

[25] Consumentenprijsindex 2019: Centraal Bureau voor de Statistiek; [Available from: https://opendata.cbs.nl/statline/#/CBS/nl/dataset/83131NED/table?ts=1536307973279.

[26] Koster L. Generieke kosten medicatiebeoordeling. KNMP; 2014.

[27] Faria R, Gomes M, Epstein D, White IR. A guide to handling missing data in cost-effectiveness analysis conducted within randomised controlled trials. Pharmacoeconomics. 2014;32(12):1157–70. doi: 10.1007/s40273-014-0193-3 25069632 PMC4244574

[28] White IR, Royston P, Wood AM. Multiple imputation using chained equations: Issues and guidance for practice. Stat Med. 2011;30(4):377–99. doi: 10.1002/sim.4067 21225900

[29] Rubin D. Inference and missing data. Biometrika. 1976;63(3):581–92.

[30] Willan AR, Briggs AH, Hoch JS. Regression methods for covariate adjustment and subgroup analysis for non-censored cost-effectiveness data. Health economics. 2004;13(5):461–75. doi: 10.1002/hec.843 15127426

[31] Bertram MY, Lauer JA, De Joncheere K, Edejer T, Hutubessy R, Kieny MP, et al. Cost-effectiveness thresholds: pros and cons. Bulletin of the World Health Organization. 2016;94(12):925–30. doi: 10.2471/BLT.15.164418 27994285 PMC5153921

[32] Bryant-Lukosius D, Carter N, Reid K, Donald F, Martin-Misener R, Kilpatrick K, et al. The clinical effectiveness and cost-effectiveness of clinical nurse specialist-led hospital to home transitional care: a systematic review. Journal of evaluation in clinical practice. 2015;21(5):763–81. doi: 10.1111/jep.12401 26135524

[33] Stamp KD, Machado MA, Allen NA. Transitional care programs improve outcomes for heart failure patients: an integrative review. The Journal of cardiovascular nursing. 2014;29(2):140–54. doi: 10.1097/JCN.0b013e31827db560 23348223

[34] van den Brink M, van den Hout WB, Stiggelbout AM, van de Velde CJ, Kievit J. Cost measurement in economic evaluations of health care: whom to ask? Medical care. 2004;42(8):740–6. doi: 10.1097/01.mlr.0000132351.78009.a1 15258475

[35] Seidl H, Meisinger C, Kirchberger I, Burkhardt K, Kuch B, Holle R. Validity of self-reported hospital admissions in clinical trials depends on recall period length and individual characteristics. Journal of evaluation in clinical practice. 2016;22(3):446–54. doi: 10.1111/jep.12506 26711475

[36] Sterne JA, White IR, Carlin JB, Spratt M, Royston P, Kenward MG, et al. Multiple imputation for missing data in epidemiological and clinical research: potential and pitfalls. BMJ (Clinical research ed). 2009;338:b2393. doi: 10.1136/bmj.b2393 19564179 PMC2714692

[37] Verweij L, Spoon DF, Terbraak MS, Jepma P, Peters RJG, Scholte Op Reimer WJM, et al. The Cardiac Care Bridge randomized trial in high-risk older cardiac patients: A mixed-methods process evaluation. J Adv Nurs. 2021;77(5):2498–510. doi: 10.1111/jan.14786 33594695 PMC8048800

