# Supplementary material for: The Cardiac Care Bridge transitional care program for the management of older high-risk cardiac patients: An economic evaluation alongside a randomized controlled trial
Source: PLoS One. 2022 Jan 27;17(1):e0263130. doi: 10.1371/journal.pone.0263130 (PMC8794155; doi:10.1371/journal.pone.0263130)
Supplement: S1 Table — (DOCX) [file pone.0263130.s002.docx]

Supplemental file 1. CHEERS checklist

| **Section/item** | **Item No** | **Recommendation** | **Reported on Page No / Line No** |
| --- | --- | --- | --- |
| **Title and abstract** |  |  |  |
| Title | 1 | Identify the study as an economic evaluation or use more specific terms such as “cost-effectiveness analysis”, and describe the interventions compared. | 1 |
| Abstract | 2 | Provide a structured summary of objectives, perspective, setting, methods (including study design and inputs), results (including base case and uncertainty analyses), and conclusions. | 2 |
| **Introduction** |  |  |  |
| Background and objectives | 3 | Provide an explicit statement of the broader context for the study. Present the study question and its relevance for health policy or practice decisions. | 3 |
| **Methods** |  |  |  |
| Target population and  subgroups | 4 | Describe characteristics of the base case population and  subgroups analysed, including why they were chosen. | 4 |
| Setting and location | 5 | State relevant aspects of the system(s) in which the decision(s) need(s) to be made. | 4, 5 |
| Study perspective | 6 | Describe the perspective of the study and relate this to the costs being evaluated. | 4, 5, 6 |
| Comparators | 7 | Describe the interventions or strategies being compared and state why they were chosen. | 5, 6 |
| Time horizon | 8 | State the time horizon(s) over which costs and consequences are being evaluated and say why appropriate. | 6, 7 |
| Discount rate | 9 | Report the choice of discount rate(s) used for costs and  outcomes and say why appropriate. | NA |
| Choice of health  outcomes | 10 | Describe what outcomes were used as the measure(s) of  benefit in the evaluation and their relevance for the type of analysis performed. | 6, 7 |
| Measurement of  effectiveness | 11a | *Single study-based estimates:* Describe fully the design  features of the single effectiveness study and why the single study was a sufficient source of clinical effectiveness data. | 4, 5, 6 |
|  | 11b | *Synthesis-based estimates:* Describe fully the methods used for identification of included studies and synthesis of clinical effectiveness data. | NA |
| Measurement and  valuation of preference  based outcomes | 12 | If applicable, describe the population and methods used to elicit preferences for outcomes. | NA |
| Estimating resources  and costs | 13a | *Single study-based economic evaluation:* Describe approaches  used to estimate resource use associated with the alternative  interventions. Describe primary or secondary research methods for valuing each resource item in terms of its unit cost. Describe any adjustments made to approximate to opportunity costs. | 9, 10 |
|  | 13b | *Model-based economic evaluation:* Describe approaches and  data sources used to estimate resource use associated with  model health states. Describe primary or secondary research  methods for valuing each resource item in terms of its unit  cost. Describe any adjustments made to approximate to  opportunity costs. | NA |
| Currency, price date,  and conversion | 14 | Report the dates of the estimated resource quantities and unit  costs. Describe methods for adjusting estimated unit costs to  the year of reported costs if necessary. Describe methods for  converting costs into a common currency base and the  exchange rate. | 7, 8 |
| Choice of model | 15 | Describe and give reasons for the specific type of decision analytical model used. Providing a figure to show model structure is strongly recommended. | NA |
| Assumptions | 16 | Describe all structural or other assumptions underpinning the  decision-analytical model. | NA |
| Analytical methods | 17 | Describe all analytical methods supporting the evaluation. This could include methods for dealing with skewed, missing, or censored data; extrapolation methods; methods for pooling  data; approaches to validate or make adjustments (such as half  cycle corrections) to a model; and methods for handling  population heterogeneity and uncertainty. | 9, 10 |
| **Results** |  |  |  |
| Study parameters | 18 | Report the values, ranges, references, and, if used, probability  distributions for all parameters. Report reasons or sources for  distributions used to represent uncertainty where appropriate.  Providing a table to show the input values is strongly  recommended. | 11, 12 |
| Incremental costs and  outcomes | 19 | For each intervention, report mean values for the main  categories of estimated costs and outcomes of interest, as well  as mean differences between the comparator groups. If  applicable, report incremental cost-effectiveness ratios. | 13, 14 |
| Characterising  uncertainty | 20a | *Single study-based economic evaluation:* Describe the effects  of sampling uncertainty for the estimated incremental cost and  incremental effectiveness parameters, together with the impact of methodological assumptions (such as discount rate, study perspective). | 14, 15, 16, 17 |
|  | 20b | *Model-based economic evaluation:* Describe the effects on the  results of uncertainty for all input parameters, and uncertainty  related to the structure of the model and assumptions. | NA |
| Characterising  heterogeneity | 21 | If applicable, report differences in costs, outcomes, or cost effectiveness that can be explained by variations between  subgroups of patients with different baseline characteristics or  other observed variability in effects that are not reducible by  more information. | 16, 17 |
| **Discussion** |  |  |  |
| Study findings,  limitations,  generalisability, and  current knowledge | 22 | Summarise key study findings and describe how they support  the conclusions reached. Discuss limitations and the  generalisability of the findings and how the findings fit with  current knowledge. | 18, 19, 20, 21 |
| **Other** |  |  |  |
| Source of funding | 23 | Describe how the study was funded and the role of the funder  in the identification, design, conduct, and reporting of the  analysis. Describe other non-monetary sources of support. | 22 |
| Conflicts of interest | 24 | Describe any potential for conflict of interest of study  contributors in accordance with journal policy. In the absence  of a journal policy, we recommend authors comply with  International Committee of Medical Journal Editors recommendations. | 22 |
