# Supplementary material for: The Cardiac Care Bridge transitional care program for the management of older high-risk cardiac patients: An economic evaluation alongside a randomized controlled trial
Source: PLoS One. 2022 Jan 27;17(1):e0263130. doi: 10.1371/journal.pone.0263130 (PMC8794155; doi:10.1371/journal.pone.0263130)
Supplement: S2 Table — (DOCX) [file pone.0263130.s003.docx]

Supplemental file 2. Variables included in the imputation model.

| **Outcome variables** | **Predictor variables^a^** | **Covariates included in the model** |
| --- | --- | --- |
| Healthcare costs (primary, secondary and informal care): baseline and 6 months follow-up | Nationality | Hospital of inclusion |
| Healthcare costs (primary and secondary care): 3 months follow-up | AUDIT-C alcohol use questionnaire, baseline | Dutch Safety Management System-score: baseline |
| HQoL, EQ5D-5L: baseline, 3 and 6 months follow-up | Index hospital admission acute | Diagnosis heart failure, acute coronary syndrome, other |
| Composite outcome readmission and mortality: 6 months  follow-up |  | Charlson Comorbidity Index: baseline |
|  |  | Age |
|  |  | Sex |
|  |  | Mini Mental State Examination: baseline |
|  |  | Living arrangement |
|  |  | Admission in six months prior to admission |

^a^Predictor variables included variables that differed between the intervention group and the usual care group at baseline, variables that were related to missingness of data and variables that were associated with the outcomes.
