# Supplementary material for: The Cardiac Care Bridge transitional care program for the management of older high-risk cardiac patients: An economic evaluation alongside a randomized controlled trial
Source: PLoS One. 2022 Jan 27;17(1):e0263130. doi: 10.1371/journal.pone.0263130 (PMC8794155; doi:10.1371/journal.pone.0263130)
Supplement: S3 Table — (DOCX) [file pone.0263130.s004.docx]

| **Healthcare utilization** | | **Missing**  Intervention | **Missing**  Control |
| --- | --- | --- | --- |
| **Primary care** | | **N (%)** | **N (%)** |
| General practitioner consultation | | 35 (23) | 37 (24) |
| Community pharmacist medication reconciliation | | 0 | 60 (39) |
| Home care | |  |  |
|  | Community nursing | 36 (24) | 37 (24) |
|  | Personal care | 36 (24) | 37 (24) |
|  | Domestic care at home | 36 (24) | 37 (24) |
| Care hotel (in nursing home) | | 32 (21) | 37 (24) |
| Day-care | | 35 (23) | 37 (24) |
| Physical therapy | | 0 | 0 |
| Physical therapy, home visit | | 0 | 0 |
| **Secondary care** | |  |  |
| Emergency room | | 0 | 0 |
| Hospital admission | | 53 (35) | 48 (31) |
| Hospital ICU admission | | 36 (24) | 37 (24) |
| Outpatient clinic | | 36 (24) | 37 (24) |
| Rehabilitation | |  |  |
|  | Institutional | 33 (22) | 37 (24) |
|  | Outpatient cardiac rehabilitation | 33 (22) | 37 (24) |
| Residential and nursing home care | | 32 (21) | 37 (24) |
| **Informal care** | |  |  |
| Voluntary care, housekeeping, practical caregiver support | | 61 (40) | 57 (37) |

Supplemental file 3. Missing data on healthcare utilization
